# White blood cell subpopulation changes and prevalence of neutropenia among Arab diabetic patients attending Dasman Diabetes Institute in Kuwait

**DOI:** 10.1371/journal.pone.0193920

**Published:** 2018-03-16

**Authors:** Fatima Ali, Faisal Alsayegh, Prem Sharma, Mohammad Waheedi, Tania Bayoud, Faisal Alrefai

**Affiliations:** 1 Faculty of Medicine, Health Science Center–Kuwait University, Al-Jabriya, Kuwait; 2 Faculty of Pharmacy, Health Science Center–Kuwait University, Al-Jabriya, Kuwait; 3 Dasman Diabetes Institute, Sharq, Kuwait; University of Texas Health Science Center at San Antonio, UNITED STATES

## Abstract

**Background:**

The effects of diabetes mellitus on the differential white blood cell count are not widely studied in the Arab populations. The objective of this cross-sectional, retrospective study is to assess the influence of chronic diabetes mellitus on white blood cell counts, absolute neutrophil (ANC) and lymphocyte counts (ALC) as well as the prevalence of benign ethnic neutropenia among Arabs attending the Dasman Diabetes Institute (DDI) in Kuwait.

**Methods and findings:**

1,580 out of 5,200 patients registered in the DDI database qualified for our study. Age, gender, HbA1c and creatinine levels, estimated glomerular filtration rate as well as average WBC, ANC and ALC levels, presence of diabetes-associated complications and anti-diabetic medications were analyzed. Our results showed the mean value of the WBC was 7.6 ± 1.93 x 10^9^/L (95% CI: 2.95–17.15). The mean ANC was 4.3 x 10^9^/L (95% CI: 0.97–10.40) and mean ALC was 2.5 x 10^9^/L (95% CI: 0.29–10.80). Neutropenia (ANC: <1.5 x 10^9^/L) was detected in fifteen patients (0.94%). Six patients (0.4%) fulfilled the definition of lymphopenia (ALC < 1 x10^9^/L). Patients with an HbA1c ≥ 7% and those taking at least 3 anti-diabetic medications showed higher values for ANC and ALC. Patients with diabetes-associated neuropathy or nephropathy displayed higher mean ANC values. Our study was limited by overrepresentation of patients over 50 years old compared to those under 50 as well as selection bias given its retrospective nature.

**Conclusions:**

Our study showed that patients with poorly controlled diabetes displayed higher ANC and ALC levels. In addition, patients with DM-associated complications showed higher ANC levels. This finding would suggest that DM exerts a pro-inflammatory influence on differential WBC counts. Our study also showed that the prevalence of benign ethnic neutropenia was lower than previously reported in other studies.

## Introduction

Diabetes mellitus (DM) is a chronic metabolic disorder characterized by persistently elevated serum blood glucose levels [1]. In the long term, poorly controlled DM is associated with secondary immunodeficiency and frequent infections, thereby contributing to patient’s morbidity and mortality [2–4]. The worldwide prevalence of DM is estimated to be 6.4% [5]. However, certain countries in the Middle East have reported much higher prevalence in their populations. This includes Kuwait (21.1%), Lebanon (20.2%), Qatar (20.2%) and Saudi Arabia (20.0%) [6]. The influence of chronic diabetes on the immune and hematological systems is the subject of ongoing research [7–10]. Data suggests that DM has a wide detrimental effect on complement function as well as both innate and adaptive immunity [2,8,9,11,12]. DM has been shown to exert an adverse effect on polymorphonuclear cells (PMN) in terms of chemotaxis, phagocytic functions and oxidative burst capabilities [2,13]. Chronically uncontrolled DM has also been shown to possibly impair T lymphocyte function, particularly CD4+ cell proliferative responses to protein antigens [14]. One study found a mild but significant neutropenia preceded and accompanied type I diabetes [15]. Another study showed an inverse relationship between white blood cell (WBC) count and insulin tolerance; hence, leukocytosis was associated with the development of DM [16–18]. The effect of DM on overall neutrophil and lymphocyte numbers within Arab populations has not been reported previously in the literature.

Benign ethnic neutropenia (BEN) is a relatively common condition throughout the Middle East, especially among those of African descent [19]. It is a clinical diagnosis based on persistent neutropenia with normal levels of other white blood cell lines, as well as the absence of secondary causes of neutropenia or hematological disorders [20–23]. There are many reports of the prevalence of BEN in Arab populations. However, the prevalence of this disorder in a diabetic Arab population is lacking. The purpose of this retrospective, cross-sectional study is to assess the effects of DM on white blood cell (WBC) subpopulations and to estimate the prevalence of benign ethnic neutropenia in a cohort of Arab patients attending the Dasman Diabetes Institute (DDI) of Kuwait.

## Materials and methods

### Study design, data source, population and study protocol approval

DDI is a leading research center for diabetes mellitus in Kuwait. It also operates a large outpatient facility dedicated to managing DM and its complications. Data on patients attending the outpatient clinics between June 2006 and February 2015 was retrospectively collected through the DDI electronic healthcare records, a network database which is routinely updated by treating physicians after each patient encounter. The patient laboratory data was retrieved via the Laboratory Information System (LIS). The data extraction for the sole purpose of research was first approved by the International Scientific Advisory Board (ISAB) at DDI and later by the DDI Ethical Review Committee (ERC) (approval number: RA 2014–040). All data was fully anonymized before access by the authors and the requirement for informed consent was waived by both the ISAB at DDI and the local ethics committee before obtaining the data. Investigators underwent the online training course “Protecting Human Research Participants”, as per National Institute of Health (NIH) requirements. DDI is a research facility and patients who attend provide informed, written consent to have their data used in all future research. Given the retrospective nature of our study, we were not required to obtain permission directly from every patient. Instead, DDI ethics committee granted our group access their database and waived the requirement to obtain informed consent. All data was anonymized and the IRB waived the requirement for informed consent.

### Inclusion and exclusion criteria

Inclusion criteria included all patients at least 18 years of age, of Middle Eastern descent, and who carried a diagnosis of either type I or type II DM for at least one year. All selected patients had active files in DDI and had undergone at least two separate assessments within a year from the end of February 2015. Exclusion criteria included patients of non-Middle Eastern descent, those under 18 years, in addition to those suffering from any known hematological, malignant or infectious conditions at the time of data collection. Patients with incomplete records or a single visit were also excluded.

### Data collection

The collected data included patient age, gender, current medications and presence of any DM-associated complications as well as laboratory data. For each patient, two most recent WBC values, including 2 absolute neutrophil counts (ANC) and absolute lymphocyte counts (ALC), were collected from the database and the average value for each population calculated. Creatinine, estimated glomerular filtration rate (eGFR) and glycated hemoglobin (HbA1c) were also collected from each patient.

### Definitions

Our study defined diabetes mellitus as an HbA1c value ≥ 7%, as defined by the American Diabetes Association [24]. Polypharmacy was defined when patients reported taking at least two or more medications as part of their diabetes management. Normal WBC range was defined as a value between 4–10 x10^9^/L [25]. For ANC, the resultant averages were categorized into 4 groups: average ANC ≥ 2 x10^9^/L, 1.5–2 x10^9^/L, ANC 1–1.5 x10^9^/L, and finally <1 x10^9^/L. Neutropenia was defined as an ANC < 1.5 [26]. For ALC, the resultant averages were also categorized into 4 groups: ALC ≥ 5 x10^9^/L, ALC 3–5 x 10^9^/L, ALC 1–3 x 10^9^/L, and finally ALC < 1 x10^9^/L. Lymphopenia was defined as an ALC < 1 x10^9^/L [27]. A creatinine value of ≥ 115 μmol/L and estimated glomerular filtration rate (eGFR) < 90 mL/min/1.73m^2^ were defined as consistent with renal impairment [28].

### Statistical analysis

The data management, analysis and graphical presentation were performed using the computer software ‘Statistical Package for Social Sciences, SPSS version 24.0’ (IBM Corp, Armonk, NY, USA). The patients' distribution according to their demographics, clinical features and laboratory findings were presented as number and percentages for each category. The quantitative or continuous variables were first ascertained for normal distribution assumption, applying the Kolmogorov-Smirnov test, and presented as; mean ± standard deviation (SD) and range for normally distributed variables, and median, range, interquartile (IQ) for skewed data. The mean values for laboratory findings were compared applying independent t-test or ANOVA, or non-parametric tests, depending on normal distribution criteria. The two-tailed probability value ‘p’ < 0.05 was considered statistically significant.

## Results

Of 5,200 registered patients at DDI, 1,580 (30.4%) met the inclusion criteria. The demographics and laboratory values are shown in Table 1. The mean creatinine and eGFR results are also shown in Table 1. Diabetes-associated complications and their relation to ANC and ALC values are shown in Table 2. Patients' reported medications and the presence of polypharmacy are displayed in Table 3. The majority (86.1%) of patients were not receiving insulin.

**Table 1:** Demographic characteristics and laboratory findings, including mean ANC* and mean ALC †, in Arab diabetic patients attending DDI (n = 1580).

Characteristics	No	%
**Gender**		
Male	794	50.3
Female	786	49.7
**Age**		
≤ 50 years	497	31.5
> 50 years	1083	68.5
Mean Age ± SD (Range)	52.3 ± 14.1 (18–71)	
**HbA1c**		
High (≥ 7.0)	1271	80.4
Low (< 7.0)	309	19.6
Mean HbA1c ± SD (Range)	8.28 ± 1.50 (4.1–14.4)	
**Creatinine**		
< 115	1408	89.7
≥ 115	162	10.3
Mean Creatinine ± SD (Range)	85.4 ± 52.6 (39–1259)	
Median (Inter-quartile)	19.7 (7.7–67.8)	
**eGFR**		
< 90	397	77.2
≥ 90	117	22.8
Mean ± SD (Range)	72.77 ± 25.49 (6.00–160.00)	
Median (Inter-quartile)	74.0 (55.0–87.5)	
**ANC**		
0.00–0.99	1	0.1
1–1.5	14	0.9
1.5–2	47	3.0
≥ 2	1492	96.0
Mean ± SD (Range)	4.28 ± 1.45 (0.97–10.40)	
Median (Inter-quartile)	4.15 (3.25–5.13)	
**ALC**		
0.00–0.99	6	0.4
1–3	1256	80.8
3–5	285	18.3
≥ 5	26	0.5
Mean ± SD (Range)	2.46 ± 0.74 (0.29–10.80)	
Median (Inter-quartile)	2.37 (1.97–2.86)	

*ANC: Absolute neutrophil count

† ALC: Absolute lymphocyte count

**Table 2:** Diabetes-associated complications and mean ANC and mean ALC in Arab diabetic patients attending DDI.

Complication		HbA1c		ANC	ALC
	N (%)	Mean ± SD	N(%)	Mean ± SD	Mean ± SD
**Neuropathy**					
Present	502 (31.8)	8.60±1.47	494 (31.7)	4.60±1.46	2.53±0.81
Absent	1078(68.2)	8.14±1.50	1060(68.3)	4.13±1.43	2.43±0.71
p-value		<0.001		<0.001	0.028
**Diabetic foot**					
Present	429 (27.2)	8.23±1.36	424(27.3)	4.40±1.56	2.45±0.71
Absent	1151(72.8)	8.30±1.55	1130(72.7)	4.23±1.41	2.47±0.76
p-value		0.337		0.057	0.555
**Retinopathy**					
Present	1033 (65.4)	8.24±1.45	1017(65.4)	4.27±1.44	2.47±0.72
Absent	547 (34.6)	8.36±1.60	537(34.6)	4.29±1.48	2.45±0.80
p-value		0.162		0.832	0.598

**Table 3:** Medications prescribed and presence of polypharmacy among diabetic patients attending DDI.

**Medicine Intake**	**No**	**Percent**
Sitagliptin	523	33.1
Aspirin	520	32.9
Metformin	374	23.7
Insulin	220	13.9
Vildagliptin	151	9.6
Liraglutide	144	9.1
Sulfonylurea	118	7.5
Clopidogrel	101	6.4
Repaglinide	88	5.6
Pioglitazone	21	1.3
Warfarin	4	0.3
**Polypharmacy**		
None	514	32.5
Single	398	25.2
Two	316	20.0
Three & More	352	22.3

**Fig 1:**
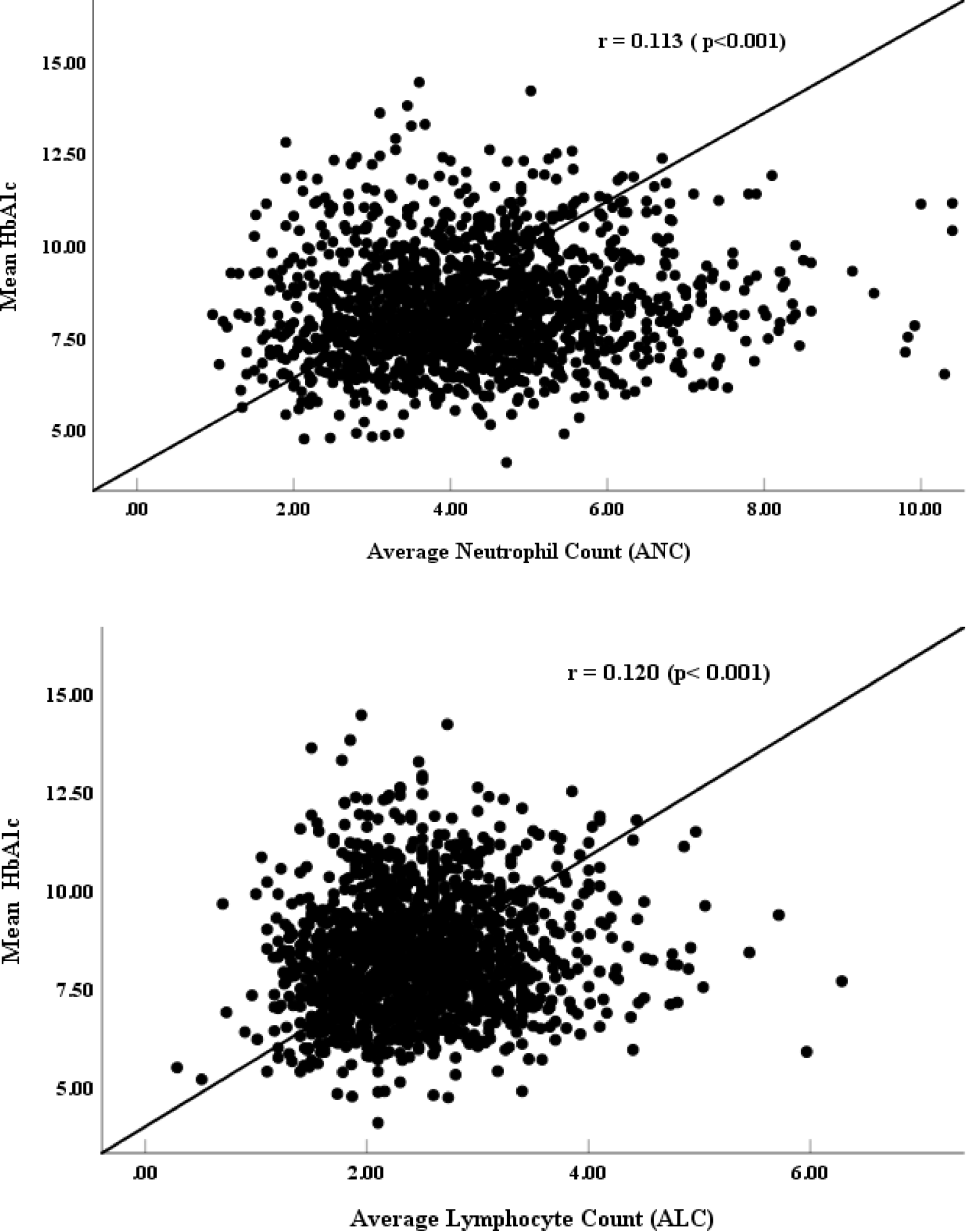
Correlation between mean HBA1C values and mean ALC and ANC among Arab diabetic patients attending Dasman Diabetes Institute.

The mean value of the WBC was 7.6 ± 1.93 x 10^9^/L (2.95–17.15). The mean ANC and ALC values are shown in Table 1. Fifteen patients (0.94%) showed an ANC value <1.5 x10^9^/L, thereby qualifying as neutropenic. Six patients had an ALC value <1 x10^9^/L, which qualified them as lymphopenic. Fig 1 shows a positive correlation between HbA1c and both ANC and ALC. The relationship between renal function (creatinine and eGFR) with ANC and ALC values are shown in Table 4. Table 5 shows an association between medications and ANC and ALC values; in particular, those on three or more medications demonstrated higher ANC and ALC values (Table 6).

**Table 4:** Differences in mean ANC and mean ALC values and patient demographics among Arab diabetic patients attending DDI.

Factor		ANC		ALC	
	N	Mean ± SD (Range)	p-value	Mean ± SD (Range)	p-value
**Gender**					
Male	780	4.25 ± 1.51 (0.97–10.40)	0.173	2.39 ± 0.76 (0.29–10.80)	<0.001
Female	774	4.31 ± 1.39 (1.05–9.13)		2.54 ± 0.72 (0.51–6.28)	
**Age**					
≤ 50 years	488	4.10 ± 1.51 (1.05–10.30)	<0.001	2.46 ± 0.75 (0.29–5.97)	0.486
> 50 years	1066	4.36 ± 1.42 (0.97–10.40)		2.47 ± 0.74 (0.70–10.80)	
**HbA1c**					
< 7.0	300	4.03 ± 1.39 (1.05–10.30)	0.001	2.32 ± 0.68 (0.29–5.97)	<0.001
≥ 7.0	1254	4.34 ± 1.46 (0.97–10.40)		2.50 ± 0.75 (0.70–10.80)	
**Creatinine**					
< 115	1390	4.22 ± 1.42 (0.97–10.40)	<0.001	2.49 ± 0.74 (0.29–10.80)	<0.001
≥ 115	161	4.75 ± 1.65 (2.00–10.40)		2.21 ± 0.70 (0.73–4.16)	
**eGFR**					
< 90	394	4.43 ± 1.42 (0.97–10.40)	0.037	2.45 ± 0.69 (0.90–5.45)	0.305
≥ 90	115	4.12 ± 1.50 (1.10–10.00)		2.54 ± 0.79 (1.05–5.05)	

**Table 5:** Medications prescribed and mean ANC and mean ALC results among Arab diabetic patients attending DDI.

Medicine		ANC		ALC	
	N	Mean ± SD	p-value	Mean ± SD	p-value
Aspirin					
Yes	515	4.46 ± 1.51	0.001	2.54 ± 0.81	0.004
No	1039	4.19 ± 1.42		2.42 ± 0.70	
Plavix					
Yes	100	4.42 ± 1.42	0.310	2.37 ± 0.76	0.227
No	1454	4.27 ± 1.45		2.47 ± 0.74	
Warfarin					
Yes	4	4.15 ± 0.54	0.669	2.76 ± 1.18	0.645
No	1550	4.28 ± 1.45		2.46 ± 0.74	
Metformin					
Yes	370	4.33 ± 1.37	0.450	2.52 ± 0.70	0.111
No	1184	4.26 ± 1.48		2.45 ± 0.75	
Sitagliptin					
Yes	516	4.42 ± 1.39	0.004	2.54 ± 0.67	0.005
No	1038	4.21 ± 1.48		2.42 ± 0.77	
Vildagliptin					
Yes	149	4.37 ± 1.35	0.390	2.51 ± 0.70	0.380
No	1405	4.27 ± 1.46		2.46 ± 0.75	
Insulin					
Yes	218	4.34 ± 1.55	0.549	2.45 ± 0.74	0.501
No	1336	4.27 ± 1.44		2.46 ± 0.74	
Sulfonylurea					
Yes	117	4.46 ± 1.51	0.155	2.61 ± 1.02	0.114
No	1437	4.26 ± 1.45		2.45 ± 0.71	
Repaglinide					
Yes	87	4.52 ± 1.41	0.097	2.68 ± 1.12	0.060
No	1467	4.26 ± 1.45		2.45 ± 0.71	
Pioglitazone					
Yes	19	4.19 ±1.59	0.808	2.49 ± 0.60	0.867
No	1535	4.28 ± 1.45		2.46 ± 0.74	
Liraglutide					
Yes	141	4.43 ± 1.32	0.152	2.63 ± 0.70	0.004
No	1413	4.26 ± 1.46		2.44 ± 0.75	

**Table 6:** Polypharmacy and mean ANC and mean ALC among Arab diabetic patients attending DDI.

		ANC		ALC	
Medicine Intake	N	Mean ± SD (Range)	p-value	Mean ± SD (Range)	p-value
None (0)	501	4.10 ± 1.46 (1.05–9.92)	<0.001*	2.37 ± 0.72 (0.29–5.97)	<0.001*
Single (1)	392	4.25 ± 1.51 (1.1–10.3)		2.43 ± 0.70 (0.73–4.86)	
Two (2)	314	4.37 ± 1.31 (1.55–10.40)	0.002(0,2) †	2.49 ± 0.67 (0.96–6.28)	0.007(0,2)
≥ Three (3)	347	4.48 ± 1.46 (0.97–10.40)	0.001(0,3) 0.012(1,3)	2.61 ± 0.85 (1.01–10.80)	0.001(0,3) 0.005(1,3)

*Non-parametric (Kruskal-Wallis test), and Mann-Whitney Test

† Comparison between none (0) and two (2), Mann-Whitney U test

## Discussion

In this retrospective study of Arab diabetic patients, the mean WBC count was 7.6 ± 1.93 x 10^9^/L. This result is comparable to another cross-sectional study of 3,772 diabetic Chinese patients, which found the mean WBC count was 7.2 ± 1.7 x 10^9^/L, although our mean is slightly higher (p<0.001). Their study also noted an association between higher WBC counts and longer disease duration, higher BMI, poorer glycemic control and lipid profile as well as increased prevalence of diabetes-associated complications [29].The role of WBC in the pathology and progression of DM is yet to be fully elucidated. WBC can act as an inflammatory marker and evidence suggests that persistently elevated counts bear a direct relationship with increased insulin resistance [16]. In addition, elevated WBC counts, even when within the normal range, may be an independent risk factor in the development of DM-associated micro- and macrovascular complications [29].

Neutrophil and lymphocyte pattern disturbances in diabetic populations are not widely available in the literature, especially with regards to Arab populations where the disease is common. Our study found 0.94% of patients had neutropenia, as defined by a persistent ANC < 1.5 x 10^9^/L. Drug-induced neutropenia was considered a possible underlying cause in those patients. A review of their medications showed some of these patients were taking a sulphonylureas and/or a meglitinide agent for glucose regulation. There are rare associations of WBC disturbances, particularly leukopenia, with these medications (< 0.1% and < 1%, respectively). However, a literature search and review of adverse drug reports revealed no associations between those two medications and neutropenia [30,31]. Therefore, drug-induced neutropenia was considered an unlikely underlying cause in our study cohort. BEN was considered as an alternative diagnosis as these patients demonstrated persistent neutropenia and their records did not indicate the presence of any infection or malignancy, thereby fulfilling the criteria for the diagnosis [19]. BEN is a clinical diagnosis [20, 21]. However, one study found a significant association between persistent neutropenia in patients of African ancestry and polymorphism in the Duffy antigen receptor for chemokines (SNP rs2814778 at chromosome 1q23.2). This study found that the null form of this variant diminished the expression of the receptor on red blood cells, and hypothesized that this variation in turn alters the concentrations of chemokines that control neutrophil production [32]. BEN is a relatively common cause of persistent neutropenia among certain ethnicities, particularly those of African and Arab descent. The population prevalence of BEN among Arabs is estimated to range from 10%– 15% [19,33], though one study from Saudi Arabia found the incidence of BEN was as high as 20% [34]. However, our study found the incidence of BEN was much lower than reported. This discrepancy may be attributed to three factors. Firstly, the reported prevalence of BEN in Saudi Arabia was based on a relatively small population study of 100 subjects which contrasts with a larger population reported in our study. Secondly, our findings may reflect the heterogeneity of the local Kuwaiti population, in that it is comprised of more varied, both Middle Eastern and non-Middle Eastern ethnicities, compared to other published reports. The close geographical proximity and historical ties between Saudi Arabia and the African continent could mean that their populations share more genetic similarities, and hence a higher prevalence of BEN, compared to the Kuwaiti population, which share greater genetic similarities with those from Asia, Africa and Europe. [35]. Thirdly, Patient selection may be another factor in the low incidence of BEN in our study. Our study exclusively comprised patients with DM, whereas other studies involved healthy populations. Diabetes in this case becomes a confounding factor, in that it establishes a low-grade inflammatory state, characterized by elevated levels of acute phase reactants such as CRP, interleukin-6 (IL-6), secretory phospholipase A_2_ and tumor necrosis factor alpha (TNFα) [36,37]. These inflammatory agents may subsequently raise neutrophil counts in patients with underlying BEN, thereby masking the true prevalence of this condition.

Our study showed patients with HbA1c ≥ 7% had a small but statistically significantly higher ANC counts compared to those with lower HbA1c. In addition, patients with DM-associated neuropathy, nephropathy and microvascular disease, represented by diabetic foot problems, showed higher ANC counts compared to patients without such complications. Our study also showed that patients on three or more anti-diabetic medications, an indication of difficult-to-control DM, had the highest ANC counts compared to patients requiring less medications. These findings suggests that poorly controlled diabetes exerts a stimulatory influence on ANC numbers. Indeed, while DM exerts a detrimental effect on almost every aspect of the innate immune system, including migratory, phagocytic, oxidative and apoptotic activities, there is also evidence to suggest that DM itself creates a pro-inflammatory milieu [7–10,37–39]. One study found that under both spontaneous and lipopolysaccharide-stimulated conditions, neutrophils from type 2 diabetic patients produced higher amounts of the cytokines interleukin-8 (IL-8), interleukin -1β (IL-1β), TNFα and interleukin-1 receptor antagonist (IL-1RA) compared to healthy controls [38]. In addition, DM has been shown to be associated with elevated CRP levels, NF-κB p65 activity, and soluble adhesion molecules such as ICAM, VCAM and E-selectin [39–41]. It is therefore possible that chronic uncontrolled diabetes creates a pro-inflammatory state causing persistent elevation of neutrophils.

Similar to ANC results, our study showed that elevated HbA1c levels were associated with a small but statistically significant increase in ALC numbers. Patients on three or more anti-diabetic medications were also found to have higher ALC numbers compared to patients on less medications. These findings further suggests that uncontrolled chronic diabetes establishes a pro-inflammatory state. However, our study did not find a significant relationship between DM-associated complications and ALC numbers.

The limitations of our study include an overrepresentation of patients over age 50 years compared to younger patients. In addition, our data does not provide the number of years since patient diagnosis, thus precluding our study's ability to explore the role of the chronicity of DM on WBC disturbances. Furthermore, given the retrospective nature of this study, selection bias and information bias are two confounding factors which may influence our results.

## Conclusion

Poorly controlled diabetes mellitus, as reflected by elevated HbA1c values and disease complications, is associated with a small but statistically significant elevation of ANC and ALC values. This elevation may reflect an underlying low-grade pro-inflammatory state established by uncontrolled DM. In addition, our study indicates that the prevalence of neutropenia among the diabetic population is lower than what has been previously reported in other Middle Eastern countries and Africa. This finding may be due to patient selection and as well population size.
